# Adrenal indeterminate nodules: CT-based radiomics analysis of different machine learning models for predicting adrenal metastases in lung cancer patients

**DOI:** 10.3389/fonc.2024.1411214

**Published:** 2024-11-12

**Authors:** Lixiu Cao, Haoxuan Yang, Huijing Wu, Hongbo Zhong, Haifeng Cai, Yixing Yu, Lei Zhu, Yongliang Liu, Jingwu Li

**Affiliations:** ^1^ Department of Nuclear Medical Imaging, Tangshan People’s Hospital, Tangshan, Hebei, China; ^2^ Department of Urology, The Second Hospital of Hebei Medical University, Shijiazhuang, Hebei, China; ^3^ Department of MRI, Tangshan People’s Hospital, Tangshan, Hebei, China; ^4^ Department of Oncology Surgery, Tangshan People’s Hospital, Tangshan, Hebei, China; ^5^ Department of Radiology, The First Affiliated Hospital of Soochow University, Suzhou, Jiangsu, China; ^6^ Department of Molecular Imaging and Nuclear Medicine, Tianjin Medical University Cancer Institute and Hospital, National Clinical Research Center for Cancer, Tianjin Key Laboratory of Cancer Prevention and Therapy, Tianjin’s Clinical Research Center for Cancer, Tianjin, China; ^7^ Department of Neurosurgery, Tangshan People’s Hospital, Tangshan, Hebei, China

**Keywords:** adrenal indeterminate nodules, radiomics, different machine learning algorithms, adrenal metastases, lung cancer

## Abstract

**Objective:**

There is a paucity of research using different machine learning algorithms for distinguishing between adrenal metastases and benign tumors in lung cancer patients with adrenal indeterminate nodules based on plain and biphasic-enhanced CT radiomics.

**Materials and Methods:**

This study retrospectively enrolled 292 lung cancer patients with adrenal indeterminate nodules (training dataset, 205 (benign, 96; metastases, 109); testing dataset, 87 (benign, 42; metastases, 45)). Radiomics features were extracted from the plain, arterial, and portal CT images, respectively. The independent risk radiomics features selected by least absolute shrinkage and selection operator (LASSO) and multivariate logistic regression (LR) were used to construct the single-phase and combined-phase radiomics models, respectively, by support vector machine (SVM), decision tree (DT), random forest (RF), and LR. The independent clinical-pathological and radiological risk factors for predicting adrenal metastases selected by using univariate and multivariate LR were used to develop the traditional model. The optimal model was selected by ROC curve, and the models’ clinical values were estimated by decision curve analysis (DCA).

**Results:**

In the testing dataset, all SVM radiomics models showed the best robustness and efficiency, and then RF, LR, and DT models. The combined radiomics model had the best ability in predicting adrenal metastases (AUC=0.938), and then the plain (AUC=0.935), arterial (AUC=0.870), and portal radiomics model (AUC=0.851). Besides, compared to clinical-pathological-radiological model (AUC=0.870), the discriminatory capability of the plain and combined radiomics model were further improved. All radiomics models had good calibration curves and DCA showed the plain and combined radiomics models had more optimal clinical efficacy compared to other models, with the combined radiomics model having the largest net benefit.

**Conclusions:**

The combined SVM radiomics model can non-invasively and efficiently predict adrenal metastatic nodules in lung cancer patients. In addition, the plain radiomics model with high predictive performance provides a convenient and accurate new method for patients with contraindications in enhanced CT.

## 1 Introduction

Lung cancer is one of the leading causes of cancer-related deaths worldwide, and early diagnosis, precise staging, and personalized treatment have consistently been the focus of medical research (1–3). The high mortality rate may be associated with the development of metastasis, and the adrenal glands is a frequent site of metastatic spread (4). Adrenal metastasis is the second most common tumor after adrenal adenoma and also the most common malignancy of the adrenal gland (5). The most common primary malignant tumors of adrenal metastasis are lung cancer (39%), and then breast cancer (35%), malignant melanoma and so on (6, 7). With the widespread application of CT, MRI, and 18F-FDG PET/CT in the diagnosis, staging, and follow-up of malignant tumors, adrenal metastases are increasingly discovered incidentally (8). While approximately 50% of incidentalomas were metastases in lung cancer patients (9). The presence of adrenal metastasis influences the treatment of lung cancer, and further evaluation is usually required, especially in lung cancer patients with no other sites of metastases except for the adrenal gland. Thus, precise qualitative diagnosis of adrenal incidentalomas in lung cancer patients during staging or follow-up is crucial for guiding treatment and predicting prognosis.

Based on clinical symptoms, endocrine function tests, and typical imaging features, many patients can obtain a specific diagnosis of adrenal lesions. However, when adrenal incidentalomas are solitary, nonfunctioning, hyperattenuating (plain CT values>10HU) nodules (long diameter(LD) ≤ 3cm) (10, 11), immediately making an accurate diagnosis of metastatic nodules based on initial abdominal or chest biphasic-enhanced CT without additional diagnostic steps remains a challenge and focus for clinicians and radiologists, especially in lung cancer patients. Although the absolute washout rate ≥ 60% and the relative washout rate ≥ 40% used for characterizing of adrenal lesions have high specificity, sensitivity, and accuracy (12, 13). However, it is sometimes not possible to accurately differentiate adrenal benign nodules from metastases due to the overlap of absolute/relative washout rates, especially for hyperattenuating nodules (14, 15). Moreover, adrenal washout CT has certain drawbacks, such as the 15 min delayed scan is difficult to implement in daily work due to the large number of patients, the additional radiation dose and medical cost. As the most sensitive examination, the sensitivity of chemical-shift MR for hyperattenuating adenoma was only 67%. Especially for lesions with plain CT values greater than 30 HU, it is difficult to distinguish them from malignant tumors such as metastases (16–18). In addition, MRI examination is time-consuming, and some patients have contraindications for MRI examination. For PET/CT, there is a certain overlap in (18F) - fluorodeoxyglucose uptake between benign lesions and metastatic tumors (19). Additionally, PET/CT will result in greater financial and time costs for the patient. In order to achieve differential diagnosis of adrenal indeterminate nodules in lung cancer patients, invasive diagnostic methods such as biopsy would be chosen by most doctors. However, it may be necessary to take multiple samples from the tumor to determine its nature due to the heterogeneity (such as necrosis, bleeding, and calcification), especially larger tumors (20). In addition, it is sometimes difficult to obtain sufficient samples through puncture due to the hidden location of the adrenal gland (21). Lastly, biopsy may lead to unnecessary anxiety, overtreatment, and some complications in patients (22).

Therefore, radiologists and clinicians encounter a real dilemma that needs to be addressed. In clinical practice, adrenal indeterminate nodules are often identified when lung cancer patients undergo routine chest or abdominal CT scans. Unfortunately, most patients are subjected to additional examinations due to the challenges associated with diagnosing adrenal metastatic nodules using traditional imaging techniques. Consequently, there is an urgent need for an efficient, straightforward, and non-invasive method to predict adrenal metastases in lung cancer patients with adrenal indeterminate nodules based on initial biphasic-enhanced CT.

Although Cao et al. (23) suggested the diagnostic model based on traditional biphasic-enhanced CT imaging features could effectively predict adrenal metastases in lung cancer patients with adrenal indeterminate tumors. However, these traditional imaging features are assessed by radiologists with the naked eye, and the subjective influence of personal clinical experience is the main limitation of this study. As an advanced image analysis technology, Radiomics can non-invasively and objectively evaluate the heterogeneity and biological characteristics of tumors, thereby partially addressing the limitations of traditional medical imaging (24–26). Recent researches have demonstrated that CT radiomics has good differential diagnostic ability for adrenal lesions, particularly in distinguishing between benign and malignant tumors (27, 28). Furthermore, Moawad et al. (29) have indicated that a radiomics model established by random forest (RF) based on plain CT images showed good diagnostic performance for adrenal indeterminate tumors. However, a previous study found that certain texture features had limited utility, with a maximum AUC of 0.69, in differentiating adrenal metastases from benign tumors when using portal CT images in lung cancer patients (30). Moreover, most of these studies have primarily focused on the differential diagnostic value of a limited set of radiomics features derived from single-phase CT scans with relatively small sample sizes. Additionally, research specifically addressing “adrenal indeterminate nodules in lung cancer patients” remains scarce. Most importantly, the reliability, efficacy, and high accuracy of predictive models are critical factors in facilitating the success of radiomics (31). Therefore, a robust radiomics study should evaluate the differential diagnostic efficacy of models based on various machine learning algorithms to identify the optimal model (32). To our knowledge, there is a paucity of research exploring the optimal individualized model for accurately predicting adrenal metastatic nodules in lung cancer patients with indeterminate nodules based on a large sample size, multi-phase CT radiomic features, various machine learning algorithms, and comparison of diagnostic performance between radiologists and radiomics models. Such a comparison is crucial for validating the effectiveness of the radiomic models; only when the diagnostic performance of these models surpasses that of the radiologists can the advantages of radiomics be conclusively demonstrated.

**Figure 1:**
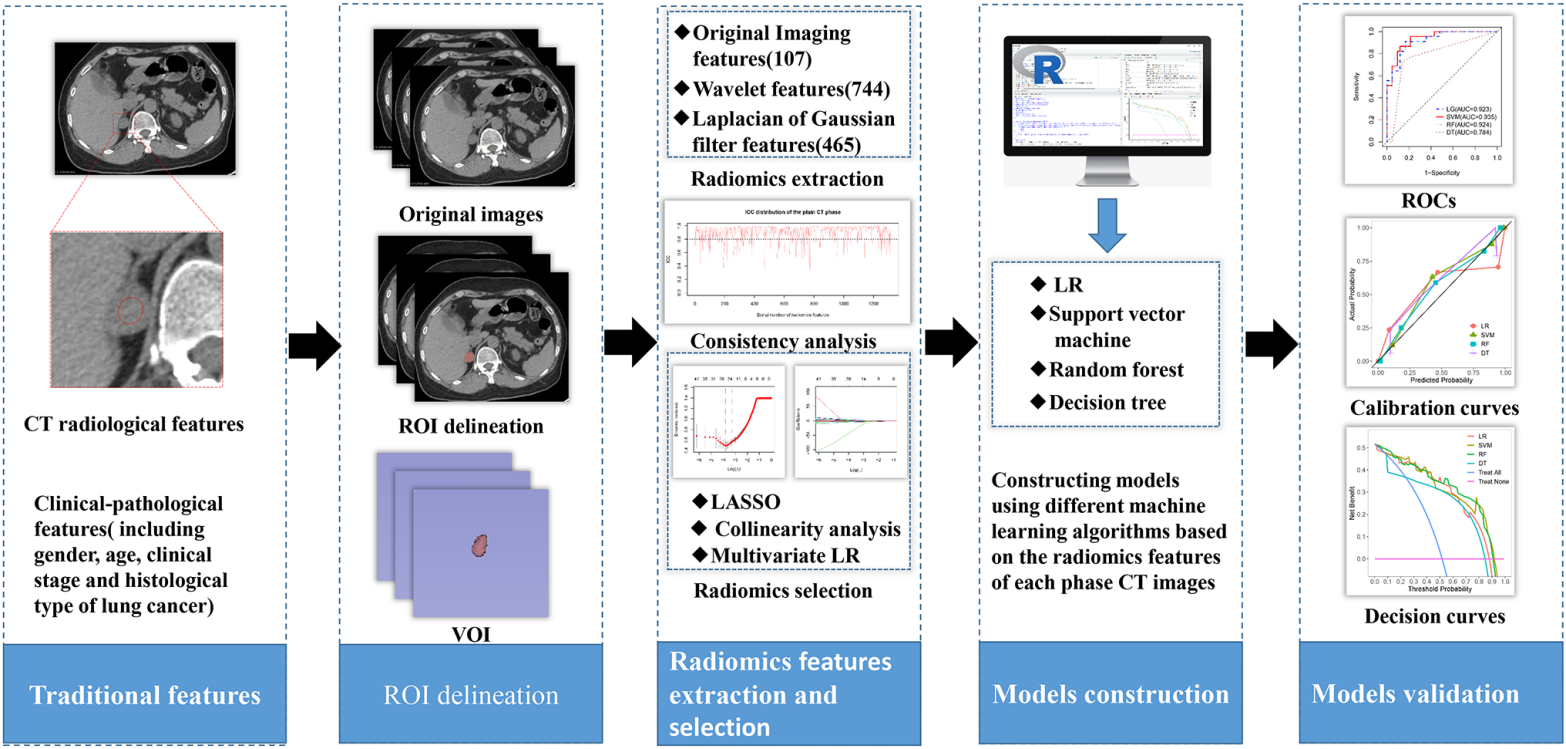
The overall workflow of the development and validation of the models.

Therefore, we chose different machine learning algorithms to develop and explore the simplest and most optimal personalized radiomics model for predicting adrenal metastases in lung cancer patients with adrenal indeterminate nodules based on initial biphasic-enhanced CT, promoting the implementation of precision medicine. The study design and pipeline are shown as **Figure 1**.

## 2 Materials and methods

### 2.1 Patients

**Figure 2:**
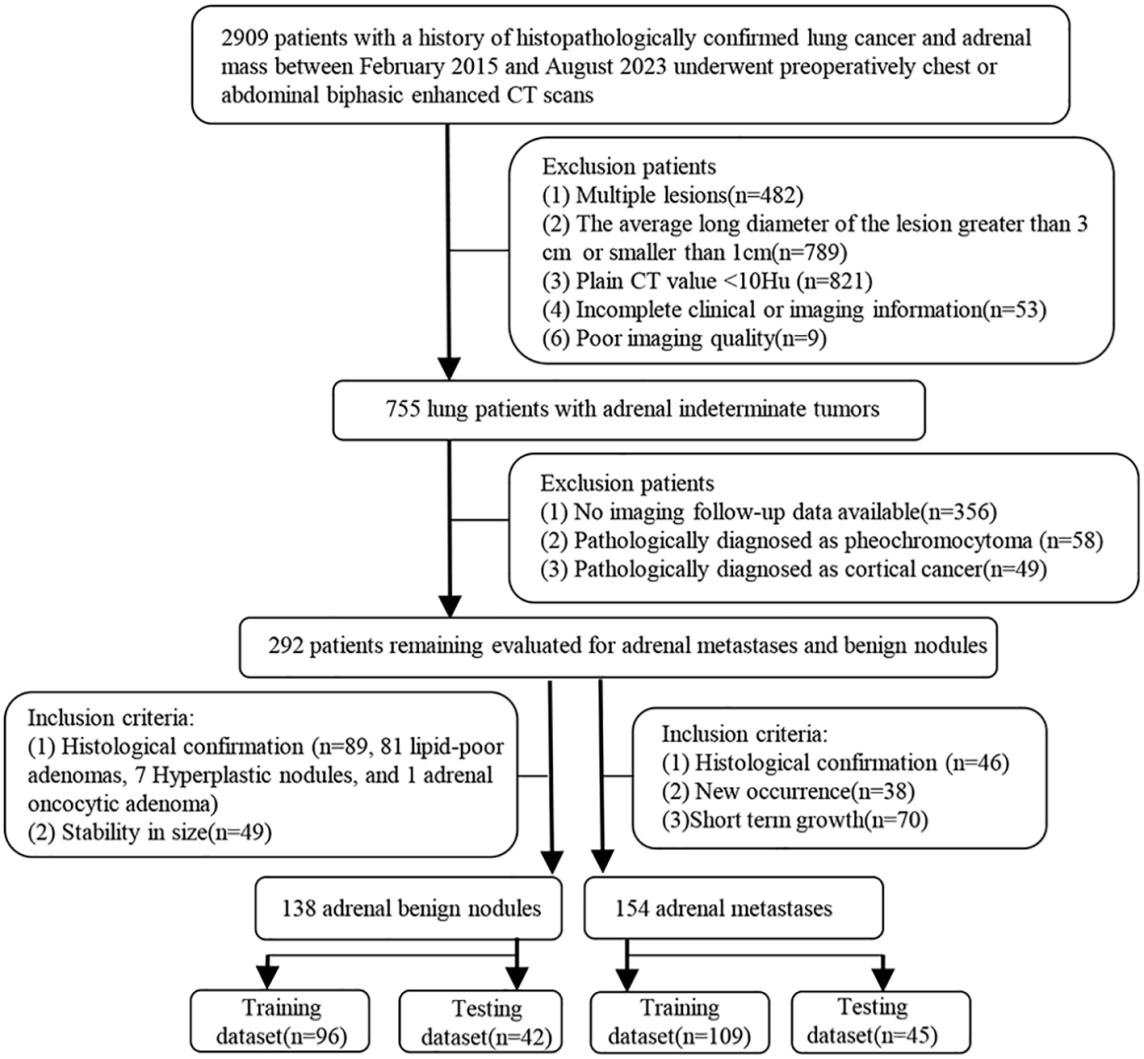
The process of dataset establishment, short time: within 6 months.

Tangshan People’s Hospital Institutional Ethics Committee approved this retrospective study and the written informed consent was obtained from each patient. The patient enrollment pathway is shown in **Figure 2**. From February 2015 to August 2023, patients histologically confirmed lung cancer, underwent abdominal or chest biphasic-enhanced CT, and with complete clinical-pathological and imaging information and adrenal indeterminate tumors: unilateral hyperattenuating (plain CT values > 10 HU) nodules (1 cm ≤ LD ≤ 3 cm) were included. The primary reasons for using biphasic-enhanced CT were: (a) lung cancer patients often accidentally discovered adrenal tumors during chest biphasic-enhanced CT. If the initial chest biphasic-enhanced CT can effectively distinguish between adrenal metastases and benign tumors, it can avoid the economic burden and radiation exposure associated with additional adrenal or abdominal enhanced CT examinations. Consequently, to standardize the inclusion criteria, we also included abdominal biphasic-enhanced CT images, excluding the delay phase. (b) previous studies have demonstrated that biphasic-enhanced CT is adequate for effective differential diagnosis of adrenal tumors (23). The main reasons for using 1cm LD as the cut-off value were: (a) allowing sufficient nodule volume for reliable quantitative measurement and (b) increasing confidence in the presence of a truly focal adrenal nodule. Additionally, patients without good image quality and imaging follow-up, or those diagnosed pathologically with cortical cancer or pheochromocytoma were excluded. Finally, 154 patients were included in the metastases group and the inclusion criteria were: (1) histologically confirmed (n =46), (2) during lung cancer treatment, the volume of adrenal nodules in the same patient increased or decreased by 20% within 6 months (33) (n = 70), and (3) newly developed adrenal lesions during follow-up (n = 38). 138 patients conformed to the inclusion criteria for benign tumors: (1) histologically confirmed (n = 89) and (2) during lung cancer treatment, the size of adrenal nodules did not change at least 1year interval (34) (n = 49). To test the performance of the diagnostic model, we randomly assigned patients to the training dataset (n = 205; 96 benign nodules, 109 metastases) and testing dataset (n = 87; 42 benign nodules, 45 metastases) at a 7:3 ratio.

### 2.2 Image protocol

This research employed two CT scanners (GE Discovery CT 750 HD and Philips Ingenuity core 64) owing to retrospective analysis. All patients first undergone abdominal or chest plain CT scan, followed by intravenous injection of 80-100 ml of nonionic iodinated contrast agent (350 mg I/ml) at a rate of 3.5 ml/s using a power injector. Then arterial (approximately 25-30 s) and portal (approximately 60-70 s) biphasic-enhanced CT scans were performed. The scanning parameters of the Philips Ingenuity core 64 were as follows: automatic tube current modulation,120 kV, matrix of 512 × 512, standard soft tissue window, 2 mm slice thickness and 2 mm slice interval, no reconstruction. The scanning parameters of the GE Discovery CT 750 HD were as follows: automatic tube current modulation,120 kV, matrix of 512 × 512, standard soft tissue window, 5 mm slice thickness and 5 mm slice interval, and reconstruction 1.25 mm slice thickness and 1.25 mm slice interval.

### 2.3 Traditional clinical-pathological-radiological model

Two radiologists with 4 and 8 years of abdominal CT experience evaluated the baseline clinical-pathological and radiological features, respectively. When there was a disagreement, a consensus was reached through discussion. The clinical-pathological and radiological features included gender, age, clinical stage and histological type of lung cancer, and the size, shape, location, CT values, cystic degeneration/necrosis, peak enhancement phase and enhanced ratio of adrenal nodules. The traditional clinical-pathological-radiological model was constructed based on the independent risk factors for predicting adrenal metastases screened by univariate logistic regression (LR) and multivariate LR.

### 2.4 Radiomics feature extraction and selection

**Figure 3:**
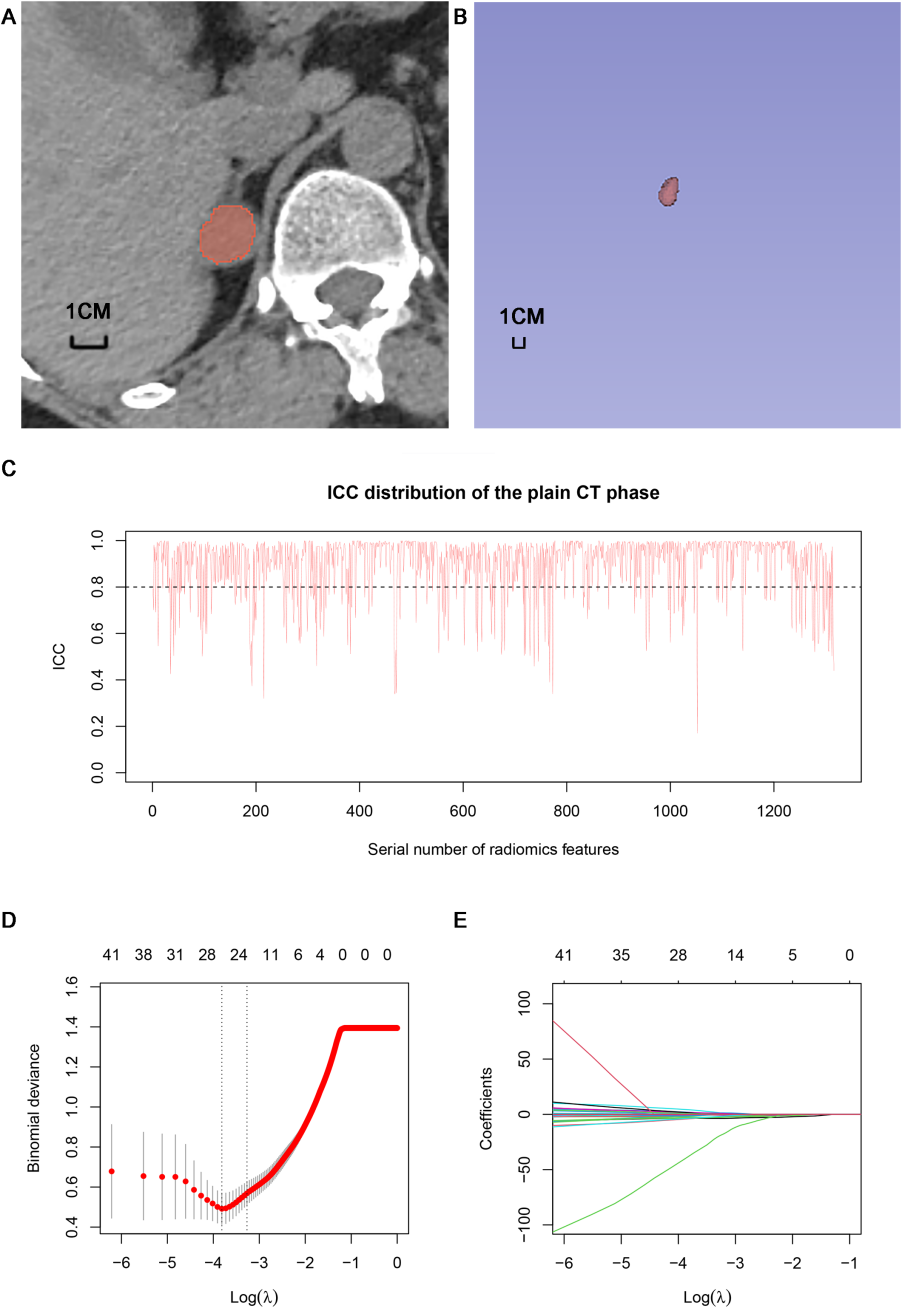
Delineation of VOI and selection of radiomics features: **(A)** Delineation of intratumoral region in the plain CT images. **(B)** Three dimensional VOI of adrenal tumors. **(C)** ICC distribution of the plain CT phase. **(D)** Binomial error graph of LASSO. **(E)** Coefficients path diagram of LASSO. VOI, volume of interest; ICC, intra-class correlation coefficient; LASSO, least absolute shrinkage and selection operator.

The volume of interests (VOIs) of adrenal nodules were delineated by two radiologists with 4 and 7 years of experience in using 3D Slicer (version 4.13.0, https://www.slicer.org) based on CT images from each phase (including unenhanced, arterial and portal), respectively. VOIs should encompass as much lesion as possible, while being careful to avoid extratumoral structures (**Figure 3A**). An open-source software (PyRadiomics, version 2.2.0) was used to process and extract radiomics features, which could provide standardized algorithms to improve data reproducibility. The calculations of radiomics features can be performed on the original or pre-processed images using wavelet and Laplacian of Gaussian filter. Radiomics features were extracted from the VOIs of adrenal indeterminate nodules (**Figure 3B**), including shape features, first-order features, second-order features (texture features), and higher-order features. Based on randomly selected 40 patients in each phase images, intra-class correlation coefficients (ICCs) were used to evaluate the interobserver reproducibility of feature extraction, respectively. ICC<0.5 meaned low consistency, 0.5-0.79 middle, and ≥ 0.8 high. Finally, the radiomics characteristics with good stability (ICC≥ 0.8) were used for subsequent analysis (**Figure 3C**). The dimensionality and redundancy of radiomics features was reduced by using the least absolute shrinkage and selection operator(LASSO) in the training dataset. The value of the penalty parameter lambda (λ) was selected through fivefold cross-validation to maintain the model’s loss within one standard error of the minimum (**Figures 3D, E**). Lastly, useful radiomics features with non-zero coefficients were selected from the plain, arterial, and portal CT images, respectively.

### 2.5 Radiomics models construction and validation

The useful features with high collinearity in the plain, arterial, and portal phases were deleted, respectively, and then the independent risk features were screened by using multivariate LR. In order to select the classifier model with the highest recognition of adrenal metastasis data, single-phase and combined-phase radiomics models were constructed and validated by using four machine learning algorithms, including decision tree(DT), logistic regression (LR), random forest (RF), and support vector machine (SVM). In addition, a clinical-pathological-radiomics model was developed by incorporating combined radiomics and the independent risk factors of clinical-pathology. Area under the receiver operating characteristic(ROC) curve(AUC), sensitivity, and specificity were used to evaluate the performance of the models in both the training and testing datasets. The comparison of AUCs between models was achieved through DeLong analysis, and the clinical value of each model was evaluated through decision curve analysis (DCA). Then, the best optimal personalized radiomics model was selected.

### 2.6 Statistical analysis

Statistical analysis and model construction were implemented by R software (version 4.2.1, http://www.rproject.org). The differences of categorical variables between two groups were compared using Fisher’s exact test or chi square. The differences of level variables such as clinical staging of lung cancer between two groups were compared using rank sum test. The differences of continuous variables between two groups were compared using Mann Whitney U-test. ICC and Kappa coefficients were used to evaluate the inter-observer reproducibility of the clinical-pathological and imaging features. Highly collinear variables (correlation coefficient>0.7) were automatically found using the “caret” package and then were deleted. LASSO was implemented using the “glmnet” package. The independent risk factors were screened using LR (“rms” package). The “rms” package “, “e1071” package “, “randomForest” package “, and “rpart” package “ were used to implement LR, SVM, RF, and DT algorithms, respectively. The “repoertROC” package was used to extract parameters for each ROC, including AUC (95% confidence interval (CI)), specificity, and sensitivity. The “caret” package was used to create a confusion matrix and extract parameters including precision, recall and F1 score. The “pROC” package was used to implement Delong test. The “ggplot2” package was used to draw calibration and decision curves. P value < 0.05 was statistically significant.

## 3 Results

### 3.1 Clinical-pathological-radiological model

**Figure 4:**
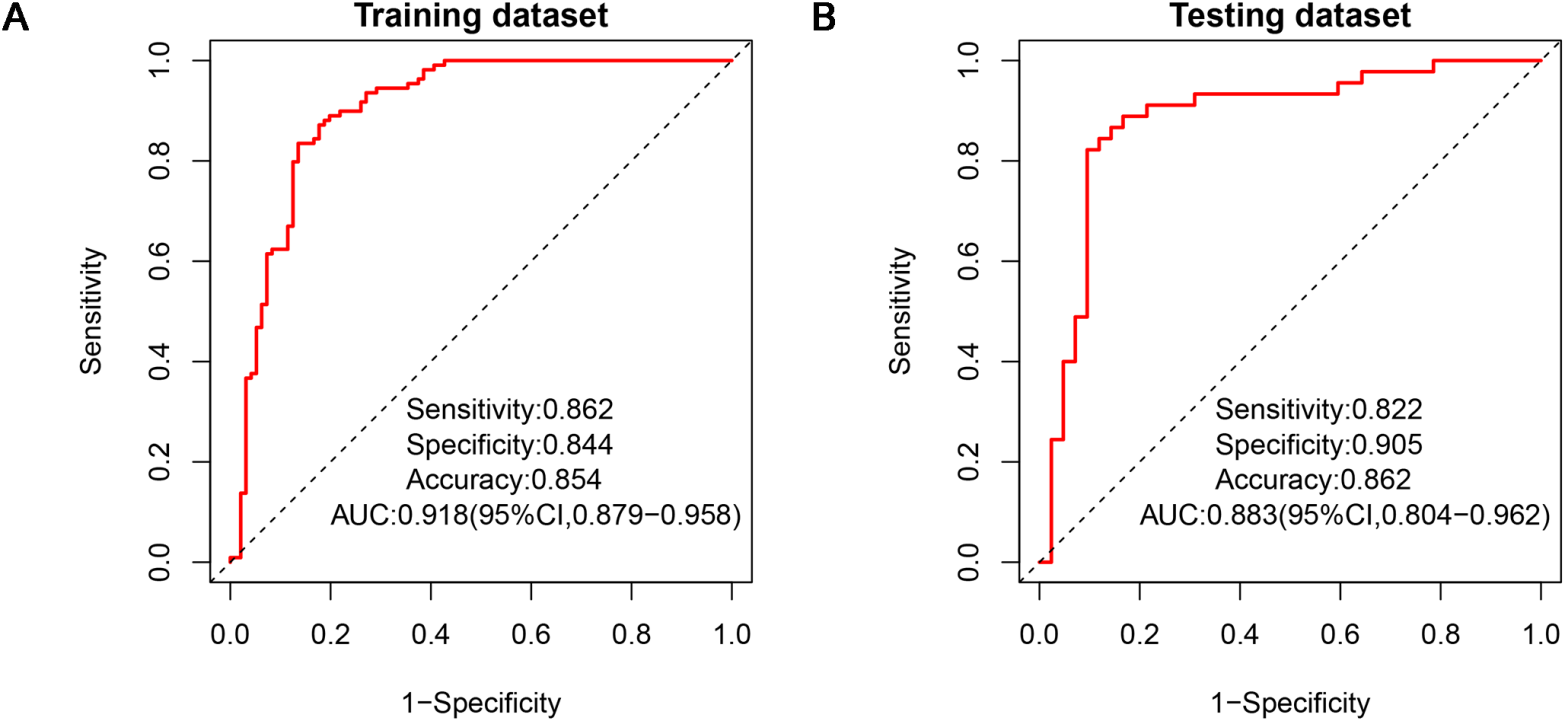
ROCs of clinical-pathological-radiological model in the training dataset **(A)** and testing dataset **(B)**. ROC, receiver operating characteristic.

Two diagnostic physicians showed good consistency in the analysis of each traditional clinical-pathological and radiological characteristics of biphasic-enhanced CT (**Supplementary Table S1**). There was no significant difference in parameters between the testing and training datasets (P>0.05), indicating the random grouping of total data was reasonable (**Table 1**). Age, gender, clinical stage and histological type of lung cancer, plain CT value, arterial enhancement rate, portal enhancement rate, and peak enhancement phase were all statistically significant between the two groups both in the training and testing datasets (P<0.05) (**Table 1**). Only arterial enhancement rate was remained for subsequent analysis due to high collinearity between arterial enhancement rate and portal enhancement rate (**Supplementary Figure S1**). And then gender, age, clinical stage of lung cancer, plain CT value, and peak enhancement phase were independent risk factors for predicting adrenal metastases screened by multivariate LR (**Supplementary Table S2**). The clinical-pathological-radiological model constructed by above five risk factors showed an AUC of 0.918 [95% CI: 0.879-0.958], a sensitivity of 0.864, a specificity of 0.844, and an accuracy of 0.854 in the training dataset, respectively; While the AUC, sensitivity, specificity, and accuracy were 0.883 [95% CI: 0.804-0.962], 0.822, 0.905, and 0.862 in the testing dataset, respectively (**Figure 4**).

**Table 1:** Clinical-pathological and radiological features of all the patients.

Parameters	Training dataset (n=205)		*P* value	Testing dataset (n=87)		*P* value	*P* ^+^ value
	Benign (n=96)	Metastases (n=109)		Benign (n=42)	Metastases (n=45)		
**Age(year)**	**58.0[48.0;64.0]**	**61.0[55.0;66.0]**	**0.002**	**53.5[44.2;64.5]**	**62.0[55.0;67.0]**	**0.013**	0.653
**Gender**			**<0.001**			**<0.001**	0.826
Female	57(59.4%)	30(27.5%)		26(61.9%)	9(20.0%)		
Male	39(40.6%)	79(72.5%)		16(38.1%)	36(80.0%)		
Long diameter (cm)	1.90[1.30;2.40]	1.90[1.50;2.30]	0.885	1.80[1.30;2.40]	2.20[1.50;2.70]	0.119	0.626
Short diameter (cm)	1.50[1.20;1.90]	1.40[1.20;1.90]	0.498	1.40[1.10;1.80]	1.60[1.20;2.00]	0.185	0.988
Location			0.293			0.072	0.834
Left	65(67.7%)	65(59.6%)		32(76.2%)	25(55.6%)		
Right	31(32.3%)	44(40.4%)		10(23.8%)	20(44.4%)		
Shape			0.176			0.120	0.421
Regular	90(93.7%)	95(87.2%)		39(92.9%)	36(80.0%)		
Irregular	6(6.3%)	14(12.8%)		3(7.1%)	9(20.0%)		
Calcification			0.624			1.000	1.000
No	95(98.9%)	106(97.3%)		41(97.6%)	44(97.8%)		
Yes	1(1.1%)	3(2.7%)		1(2.4%)	1(2.2%)		
Cystic degeneration/necrosis			0.148			0.143	0.736
No	84(87.5%)	86(78.9%)		37(88.1%)	33(73.3%)		
Yes	12(12.5%)	23(21.1%)		5(11.9%)	12(26.7%)		
**Plain CT value (HU)**	**25.00[20.00;31.20]**	**37.00[32.00;42.00]**	**<0.001**	**26.00[19.00;36.80]**	**38.00[32.00;42.00]**	**<0.001**	0.549
Arterial CT value (HU)	66.00[52.00;80.20]	64.00[53.00;77.00]	0.925	61.00[51.20;87.80]	63.00[50.00;74.00]	0.437	0.428
Portal CT value (HU)	73.50[60.00;86.20]	74.00[61.00;87.00]	0.611	77.50[63.50;95.80]	70.00[60.00;82.00]	0.103	0.959
**Arterial enhancement rate**	**1.49[0.92;2.15]**	**0.69 [0.43;1.12]**	**<0.001**	**1.44[1.02;2.00]**	**0.57[0.43;0.81]**	**<0.001**	0.431
**Portal enhancement rate**	**1.87[1.16;2.58]**	**0.89[0.71;1.39]**	**<0.001**	**1.82[1.27;2.86]**	**0.79[0.65;1.03]**	**<0.001**	0.468
**Peak enhancement phase**			**<0.001**			**<0.001**	0.639
Arterial phase	23(23.9%)	3(2.7%)		9(21.4%)	1(2.2%)		
Portal phase	54(56.3%)	66(60.6%)		29(69.1%)	27(60.0%)		
Equally enhanced	19(19.8%)	40(36.7%)		4(9.5%)	17(37.8%)		
**Clinical staging of lung cancer**			**<0.001**			**<0.001**	0.463
I	15(15.6%)	2(1.8%)		10(23.8%)	1(2.2%)		
II	24(25.0%)	6(5.5%)		6(14.3%)	2(4.4%)		
III	33(34.4%)	42(38.5%)		16(38.1 %)	16(35.6%)		
IV	24(25.0%)	59(54.2%)		10(23.8 %)	26(57.8%)		
**Histological types of lung cancer**			**<0.001**			**0.006**	0.335
Small cell	7(7.3%)	35(32.1%)		5(11.9%)	18(40.0%)		
Non small-cell	89(92.7%)	74(67.9%)		37(88.1%)	27(60.0%)		

The bold values indicate the statistically significant parameters.

### 3.2 Single-phase radiomics models using different machine learning algorithms

**Figure 5:**
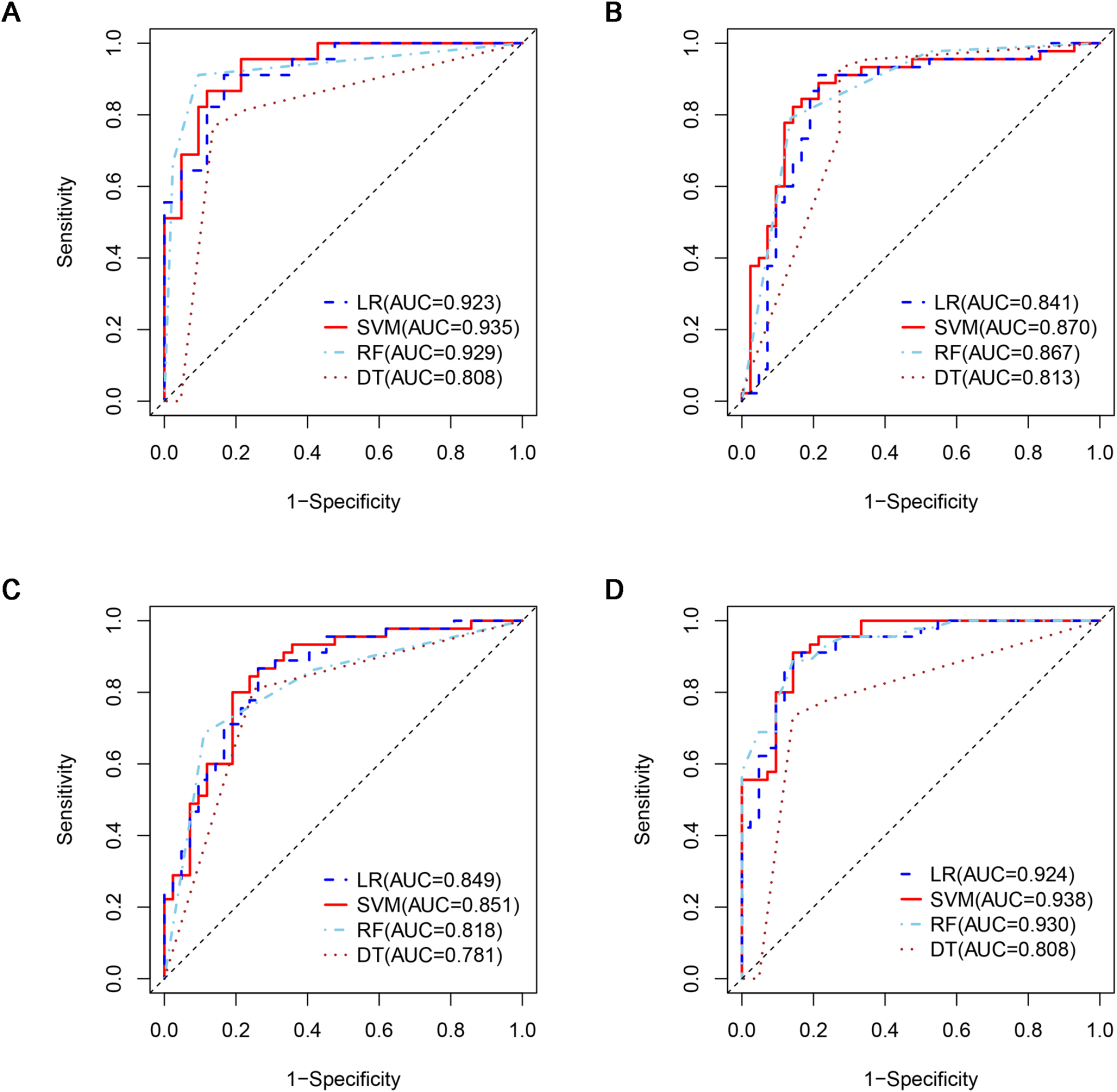
ROCs of four machine learning models in the testing dataset with different phases: **(A)**, plain phase; **(B)**, artial phase; **(C)**, portal phase; **(D)**, combined phase. ROC, receiver operating characteristic.

**Figure 6:**
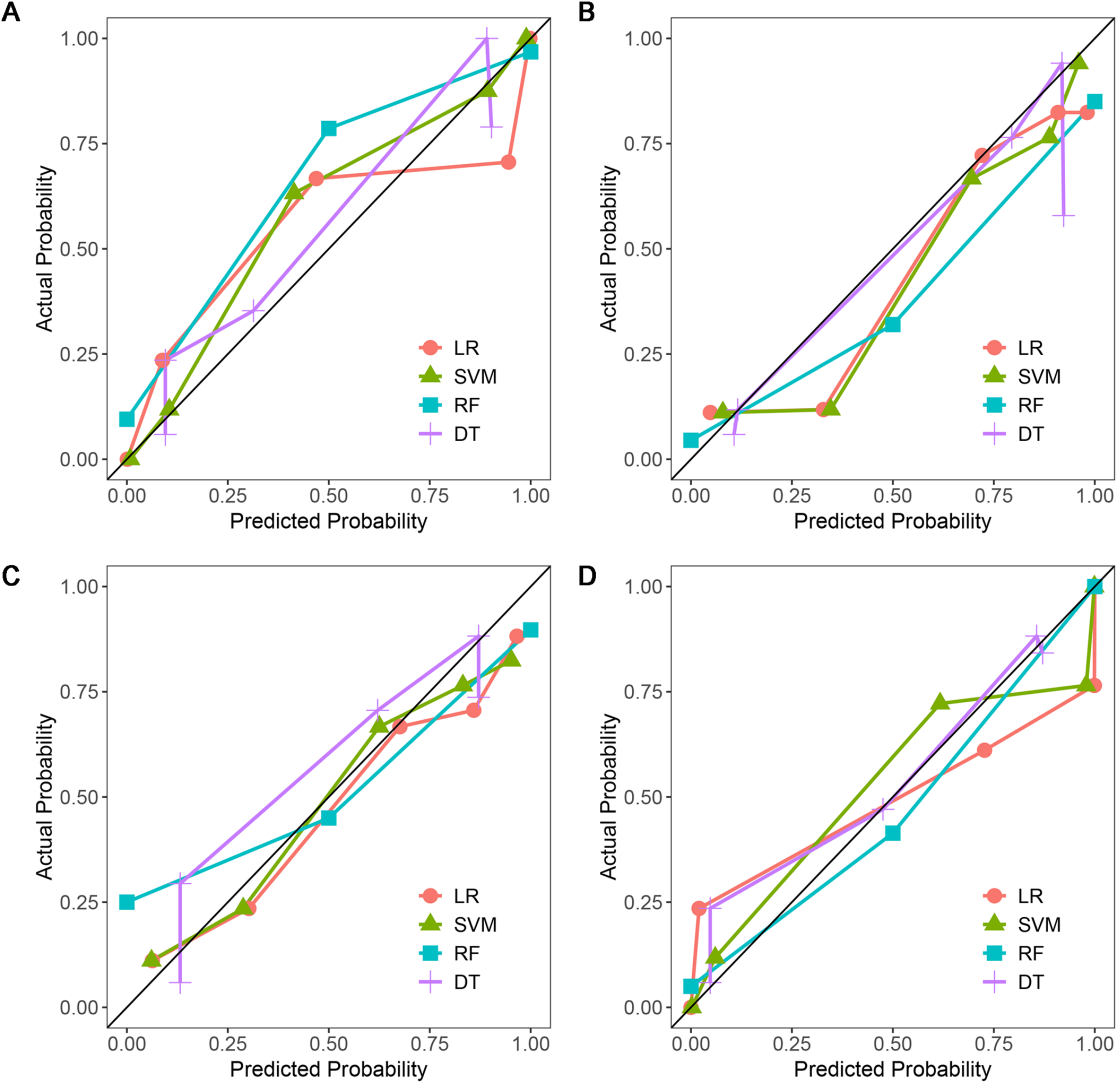
Calibration curves of four machine learning models in the testing dataset with different phases: **(A)**, plain phase; **(B)**, artial phase; **(C)**, portal phase; **(D)**, combined phase.

**Figure 7:**
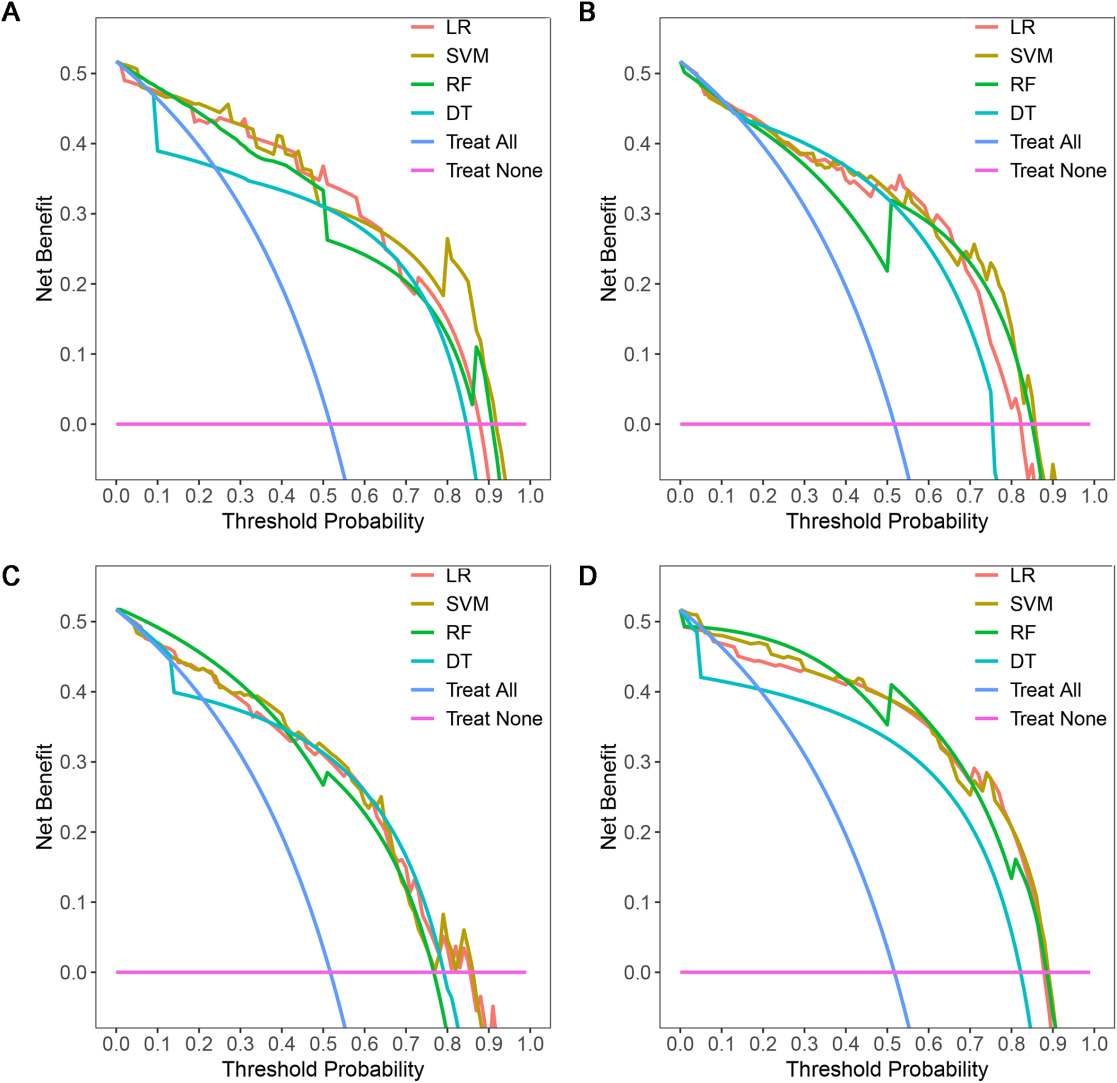
DCAs of four machine learning models in the testing dataset with different phases: **(A)**, plain phase; **(B)**, artial phase; **(C)**, portal phase; **(D)**, combined phase. DCA, decision curve analysis.

A total of 1316 radiomics features were extracted from the plain, arterial, and portal CT images, respectively. 1088 plain features, 1012 arterial features, and 983 portal features with ICC ≥ 0.8, respectively, were used for subsequent analysis. And the average ICCs of the plain features, arterial features, and portal features were 0.909, 0.889, and 0.881, respectively. A total of 22 plain features, 10 arterial features, and 13 portal features were selected using LASSO, respectively, and then features with high collinearity were all deleted. Finally, 5 plain radiomics predictors, 4 arterial radiomics predictors, and 4 portal radiomics predictors were selected by multivariate LR, respectively (**Table 2**). The correlation coefficients between the independent risk radiomics features of each phase were all relatively low (**Supplementary Figures S2A–C**). **Table 3** showed the ROCs of different machine learning radiomics models (including SVM, RF, LR, and DT) both in the testing and training datasets. The SVM models of each phase all showed the best performance in predicting adrenal metastases of lung cancer in the testing dataset, with AUC values ranging from 0.851 to 0.938, and then the RF models, LR models, and DT models (**Table 3**; **Figures 5A–C**). In the testing dataset, the four machine learning models of each phase all showed good calibration curves (**Figures 6A–C**), and DCA showed they all had high clinical net benefits (**Figures 7A–C**).

**Table 2:** Selected radiomics features in the plain, artial, portal, and combined radiomics models.

Plain radiomics model	Artial radiomics model	Portal radiomics model	Combined radiomics model
Original shape Elongation	Original shape Flatness	Original shape Flatness	#Original shape Elongation
wavelet-LLH glcm Idn	log-sigma-5-0-mm-3D firstorder InterquartileRange	log-sigma-3-0-mm-3D glszm LowGrayLevelZoneEmphasis	#wavelet-LLH glcm Idn
wavelet-LLH glcm DependenceEntropy	wavelet-LLH firstorder 90Percentile	log-sigma-4-0-mm-3D gldm SmallDependenceHighGrayLevelEmphasis	#wavelet-LLH glcm DependenceEntropy
wavelet-LHH glszm SmallAreaLowGrayLevelEmphasis	wavelet-LLH glcm Imc1	wavelet-LLH glszm GrayLevelNonUniformityNormalized	#wavelet-LHH glszm SmallAreaLowGrayLevelEmphasis
wavelet-LLL firstorder Median			#wavelet-LLL firstorder Median
			*Original shape Flatness
			*wavelet-LLH glcm Imc1

# The plain radiomics features, * The arterial radiomics features.

**Table 3:** Comparison of diagnostic efficacy for the four classification models based on different CT phase.

CT phase	algorithm	Training dataset			Testing dataset					
		AUC(95%CI)	sensitivity	specificity	AUC(95%CI)	sensitivity	specificity	precision	recall	F1 score
Plain phase	LR	0.978(0.963-0.993)	0.963	0.896	0.923(0.870-0.975)	0.911	0.833	0.854	0.911	0.882
	**SVM**	**0.977(0.961-0.992)**	**0.908**	**0.938**	**0.935(0.888-0.983)**	**0.867**	**0.881**	**0.886**	**0.867**	**0.876**
	RF	0.989(0.975-1.000)	0.982	0.958	0.929(0.875-0.983)	0.911	0.905	0.911	0.911	0.911
	DT	0.905(0.864-0.946)	0.868	0.899	0.808(0.715-0.900)	0.767	0.864	0.846	0.767	0.805
Arterial phase	LR	0.909(0.870-0.948)	0.899	0.760	0.841(0.748-0.934)	0.911	0.786	0.820	0.911	0.863
	**SVM**	**0.928(0.896-0.961)**	**0.862**	**0.865**	**0.870(0.788-0.952)**	**0.822**	**0.857**	**0.861**	**0.822**	**0.841**
	RF	0.978(0.961-0.995)	0.853	0.990	0.867(0.795-0.939)	0.791	0.7864	0.850	0.791	0.819
	DT	0.911(0.871-0.952)	0.901	0.883	0.813(0.726-0.900)	0.930	0.727	0.769	0.930	0.842
Portal phase	LR	0.903(0.864-0.943)	0.752	0.896	0.849(0.768-0.930)	0.867	0.738	0.780	0.867	0.821
	**SVM**	**0.909(0.871-0.947)**	**0.908**	**0.750**	**0.851(0.769-0.933)**	**0.800**	**0.810**	**0.818**	**0.80**	**0.809**
	RF	0.967(0.946-0.987)	0.817	0.979	0.818(0.732-0.905)	0.686	0.889	0.897	0.686	0.778
	DT	0.867(0.818-0.916)	0.891	0.832	0.781(0.688-0.873)	0.809	0.750	0.792	0.809	0.800
Combined phase	LR	0.993(0.986-1.000)	0.972	0.969	0.924(0.870-0.979)	0.867	0.881	0.886	0.867	0.876
	**SVM**	**0.992(0.983-1.000)**	**0.972**	**0.958**	**0.938(0.890-0.958)**	**0.911**	**0.857**	**0.872**	**0.911**	**0.891**
	RF	0.995(0.991-1.000)	0.954	1.000	0.930(0.887-0.974)	0.745	1.000	1.000	0.745	0.854
	DT	0.906(0.867-0.945)	0.963	0.833	0.808(0.716-0.899)	0.841	0.841	0.822	0.841	0.832

AUC, area under the receiver operating characteristic curve; CI, confidence interval; LR, logistic regression; SVM, support vector machine; RF, random forest; DT, decision tree.

The bold values indicated the SVM models of single-phase and combined-phase all showed the best performance in the testing dataset.

### 3.3 Combined-phase radiomics models using different machine learning algorithms

The independent risk factors of the combined radiomics model were identified by multivariate LR based on the radiomics predictors of each phase in the training dataset. Finally, 5 plain radiomics features and 2 arterial radiomics features were incorporated into the combined radiomics model and correlation coefficients between the independent risk features were relatively low (**Table 2**; **Supplementary Figure S2D**). **Table 3** showed the ROCs of four radiomics models both in the testing and training datasets. The SVM model showed the best performance in predicting adrenal metastases of lung cancer in the testing dataset, achieving an AUC of 0.938, along with specificity, sensitivity, precision, recall, and F1 score values of 0.911, 0.857, 0.872, 0.911, and 0.891, respectively, and then the RF model, LR model, and DT model (**Table 3**; **Figure 5D**). Additionally, the Delong test indicated that the AUC of the SVM model was significantly greater than that of the DT model; however, no significant differences were observed between the SVM model and the RF or LR models (**Supplementary Table S3**). In the testing dataset, the four machine learning models all showed good calibration curves (**Figure 6D**), and DCA showed they all had high clinical net benefits (**Figure 7D**).

### 3.4 Clinical-pathological-radiomics model

The independent risk factors of the clinical-pathological-radiomics model were identified by multivariate LR based on the radiomics predictors of each phase and the clinical-pathological predictors (gender, age, and clinical stage of lung cancer) in the training dataset. Finally, the clinical-pathological features were excluded and only the combined radiomics features were included in the clinical-pathological-radiomics model. Therefore, the clinical-pathological-radiomics model was ultimately equivalent to the combined radiomics model.

### 3.5 Comparison of the radiomics models

**Figure 8:**
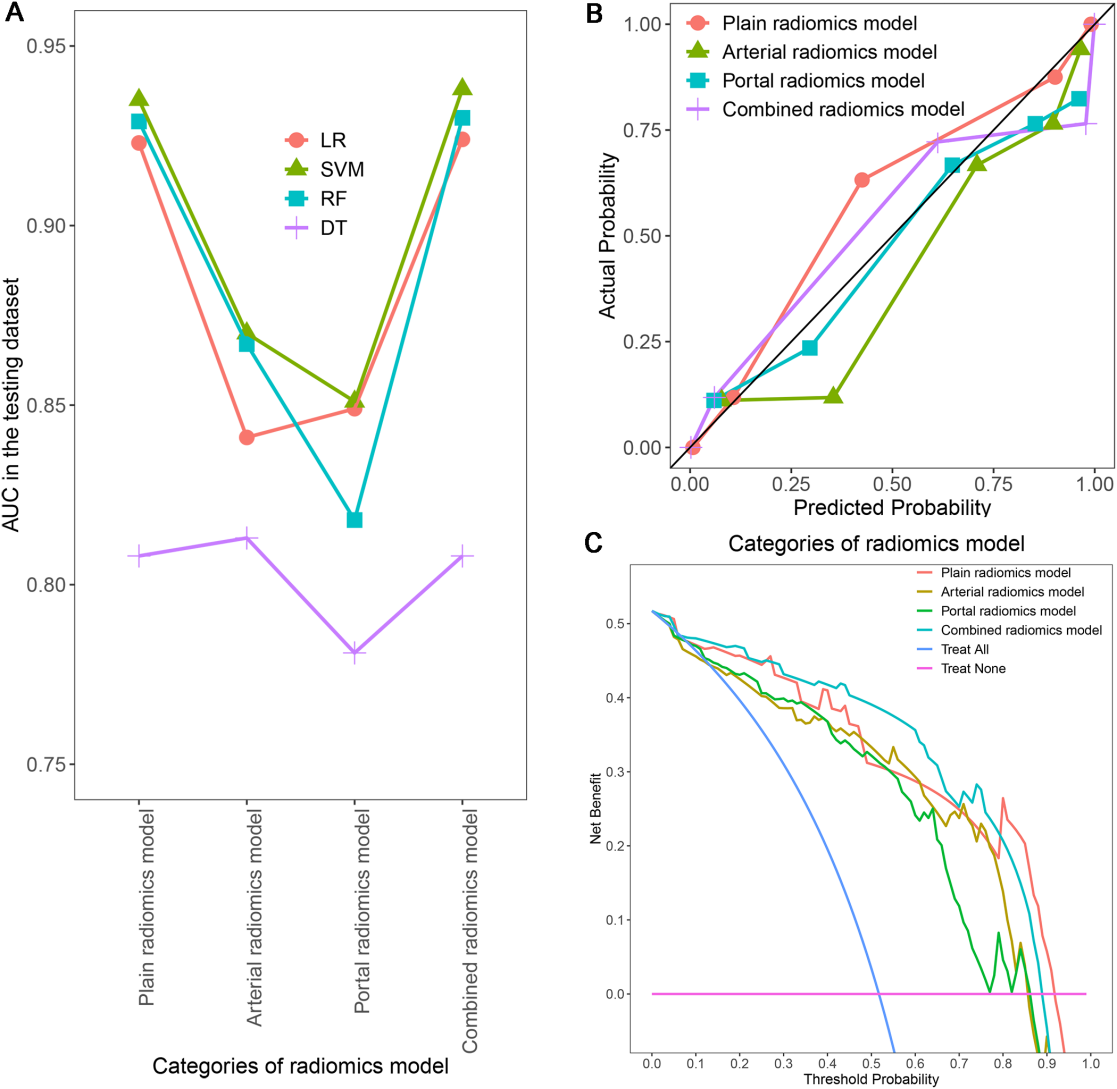
Comparison of AUCs for different radiomics models using four machine learning algorithms **(A)**; Calibration curves **(B)** and DCA **(C)** of different SVM radiomics models in the testing dataset. AUC, area under the receiver operating characteristic curve; DCA, decision curve analysis; SVM, support vector machine.

In order to ensure the sustainability and stability of radiomic models, we chose SVM models to uniformly assess the predictive performance of different radiomics models (**Figure 8A**). The predictive performance of the combined radiomics model (AUC=0.938) was highest, and then the plain radiomics model (AUC=0.935), arterial radiomics model (AUC=0.870), and portal radiomics model (AUC=0.851) in the testing dataset (**Table 4**). In addition, compared with the clinical-pathological-radiological model (AUC=0.870), the diagnostic ability of the combined radiomics model was further improved, but there was no significant difference between the two models. All the radiomics models had good calibration curves in the testing dataset (**Figure 8B**). DCA showed that the area under the curves of the plain and combined radiomics models were relatively larger than other models, and the combined radiomics model had the greatest net benefit in the probability of low risk threshold (about 0-0.8) for the testing dataset (**Figure 8C**).

**Table 4:** Comparison of AUCs between different models in the testing dataset.

Models	AUC	Z statistic	P
Combined radiomics model vs Plain radiomics model	0.938vs0.935	0.143	0.886
Combined radiomics model vs Arterial radiomics model	0.938vs0.870	1.692	0.091
Combined radiomics model vs Portal radiomics model	0.938vs0.851	2.424	0.015
Combined radiomics model vs Clinical-pathological-radiological model	0.938vs0.870	1.456	0.148

AUC, area under the receiver operating characteristic curve.

## 4 Discussion

Our study developed four radiomics models and a traditional clinical-pathological-imaging model for predicting adrenal metastatic nodules in lung cancer patients using four different machine learning algorithms based on biphasic-enhanced CT. Our results suggested SVM was the most optimal algorithm for the qualitative diagnosis of adrenal indeterminate nodules in lung cancer patients. Among all single-phase radiomics models, the plain radiomics model had the highest discriminative diagnostic performance, while the performance of combined radiomics model (including 5 plain and 2 arterial radiomics features) was further improved compared to all single-phase radiomics models and clinical-pathological-imaging model. Radiomics may provide a reliable and non-invasive method for evaluating adrenal indeterminate nodules in lung cancer patients.

In recent years, radiomics and machine learning have been already widely used for diagnosis, staging, and prognosis of tumors (35–37). Moreover, the application of radiomic analysis has been expanding within the field of adrenal lesions research. Our study has filled a gap in the literature on lung cancer patients with adrenal indeterminate nodules in the setting of CT radiomics. Ho et al. (27) found that 18 enhanced CT texture features and 9 plain CT texture features showed significant differences in distinguishing adrenal malignant and benign lesions, with an average AUC value of 0.80. However, this study analyzed only 21 second-order features and had a small sample size of just 20 patients. Winkelmann et al. (28) suggested that radiomics features derived from portal dual-energy CT could effectively distinguish between adrenal adenomas and metastases, reporting AUC values ranging from 0.89 to 0.93. Nonetheless, this study included only 32 adenomas and 17 metastases, and did not exclude lipid-rich adenomas. In contrast to these studies, our research focused specifically on adrenal indeterminate nodules in lung cancer patients to effectively predict metastases. The previous studies examined all adrenal tumors without considering tumor size and plain CT value and were not restricted to lung cancer patients. Additionally, our study had the advantage of a larger sample size encompassing all radiomics features, while the aforementioned studies had relatively small cohorts and assessed only partial radiomics features. Although Andersen et al. (30) concentrated on lung cancer patients, their study was not restricted to adrenal indeterminate nodules. And they found that the diagnostic model developed using LR based on portal CT images had limited value (the maximum AUC of 0.69) in distinguishing adrenal metastases from benign tumors. In contrast, Moawad et al. (29) focused exclusively on adrenal indeterminate tumors (LD <4cm, plain CT value >10HU, and absolute washout rate <60%), but their study was not limited to lung cancer patients. And they reported that the radiomics model established using RF based on plain CT texture features demonstrated good diagnostic performance (AUC = 0.85), with a specificity of 71.4% and a sensitivity of 84.2%. In addition, our study had the advantage of analyzing multi-phase CT radiomic features using various machine learning algorithms, while the previous studies employed single-phase CT radiomics with only one machine learning algorithm.

SVM, LR, RF, and DT are currently the most commonly used machine learning algorithms in radiomics (32, 38, 39). No one can be applicable to every medical problem among numerous algorithms. Therefore, for specific medical problem, it is necessary to compare the performance of models constructed by different machine learning algorithms in order to explore the best machine learning algorithm (32, 40). This study constructed different radiomics models using four algorithms (including SVM, LR, RF, and DT) based on enhanced CT. Our results showed SVM radiomics models of each single and combined phase had the best predictive performance in the testing dataset (AUC: 0.870-0.938), which was higher than LR model, RF model, and DT model. This indicated that SVM was the optimal algorithm for qualitative diagnosis of adrenal indeterminate tumors in lung cancer patients in our study, and SVM could be the preferred algorithm for future radiomics research on adrenal metastatic nodules. SVM is a nonlinear machine learning algorithm with the strongest generalization ability for the unknown data, which can solve high-dimensional, nonlinear, and small sample problems. It is a relatively mature machine learning algorithm (41). SVM has satisfactory stability and effectiveness, and the performance of models trained with small samples is almost the same as that of models trained with large samples (42, 43). The diagnostic ability of LR models were lower than that of SVM models, suggesting that the data of adrenal indeterminate tumors may be nonlinear or linearly indivisible. Therefore, LR models based on linear algorithms were not as effective as nonlinear SVM models (32). In addition, our study found that the radiomics model constructed by RF and DT algorithms had significantly higher AUC values in the training dataset (AUCRF range: 0.967-0.995; AUCDT range: 0.867-0.911) than in the testing dataset (AUCRF range: 0.818-0.930; AUCDT range: 0.781-0813), indicating the poor generalization ability of this models. This results suggested that models constructed by RF and DT algorithms for predicting adrenal metastatic nodules in lung cancer may exist a certain degree of overfitting, which had adverse effects on the diagnostic ability of radiomics models. This issue may be attributed to the small sample size of this study (32, 44).

The differential diagnostic efficacy of the single-phase and combined radiomics models based on the optimal algorithm SVM were further comparative analysis. This research found that the combined radiomics model included 5 plain radiomics features and 2 arterial radiomics features had the highest predictive performance for adrenal metastatic nodules of lung cancer in the testing dataset, with an AUC value of 0.938. This performance surpassed previous research results, which reported AUC values ranging from 0.69 to 0.93 (27–30). This results indicated that only the plain and arterial CT images from the initial chest or abdomen enhanced CT could reliably distinguish adrenal metastatic nodules and benign nodules in lung cancer patients by utilizing radiomics, effectively avoiding the psychological and economic pressure, and additional radiation hazards caused by unnecessary further examinations, and promoting the effective formulation of individual treatment programs. Besides, compared to 15-min delayed scan of adrenal washout CT, arterial single phase enhanced CT scan has a shorter scanning time and is easier to be widely applied. As a result, we boldly assume that 15-min delayed scan has the potential to be replaced by the arterial phase. However, this conclusion still needs further validation. A further point for concern was that the plain SVM radiomics model had the highest predictive performance in the testing dataset (AUC=0.935) among all the single phase radiomics models, slightly lower than the combined radiomics model (△ AUC=0.003). And the plain radiomics features had the greatest contribution to the combined radiomics model. This results suggested that the plain CT images may have the potential to better reflect the heterogeneity of adrenal metastatic nodules. We speculated that intra-tumoral heterogeneity may be masked by contrast agents because the blood supply of benign and metastatic adrenal nodules is relatively abundant. Feliciani (45) and Zhang (46) showed that the radiomics model based on plain CT could efficiently distinguish adrenal lipid-poor adenoma from other tumors (average AUCFeliciani=0.93, AUCZhang=0.93), indicating that plain CT radiomics features were important markers of heterogeneity in adrenal tumors, which was consistent with our study. Therefore, for the high-risk population with potential risks associated with contrast agents (such as diabetes, renal insufficiency, elderly and children), the plain radiomics model is undoubtedly the best method for safe, economical, and effective prediction of adrenal metastatic nodules in lung cancer patients.

To sum up, our findings may hold significant value for clinical practice. The primary objective of imaging in lung cancer patients with an adrenal indeterminate nodule is to differentiate between a metastatic lesion and a benign tumor. Our study has yielded very encouraging results, suggesting that further examinations may not be necessary. A plain CT scan or biphasic-enhanced CT is more readily available, offers reliable image quality, and is less expensive and time-consuming compared to adrenal contrast-enhanced CT or other imaging modalities such as MRI and PET/CT. More importantly, the initial CT images already acquired for clinical purposes may suffice for radiologists and clinicians to make accurate diagnoses based on this study. Consequently, our results could be swiftly integrated into clinical practice.

The radiomics characteristics have tight relation with the biological behavior and microstructure of lesions (47). The combined radiomics model constructed in this study included a total of 1 first-order statistical feature, 2 shape features, 1 gray level size zone matrix feature, and 3 gray level co-occurrence matrix features. The first-order statistical features mainly reflect the symmetry, uniformity, and local intensity distribution within the tumor. Shape features, in simple terms, are used to describe geometric features (48). The gray level size zone matrix features and the gray level co-occurrence matrix features can both reflect the heterogeneity and complexity of tumors from a microscopic perspective. The former mainly focuses on the texture, complexity, and clarity of lesion images, while the latter mainly focuses on the non-uniformity of grayscale levels and the variability of size regions (49–52). On the other hand, the combined radiomics model incorporated 5 wavelet transform features and 2 original features, showing that the preprocessed image features are more stable than the original image features (40).

There is another issue that requires our attention. As is well known, traditional radiological features are evaluated by radiologists using the naked eye, which can be subjectively influenced by their personal clinical experience. Additionally, radiologists tend to diagnose malignancy uncertain, relying on the likelihood of malignancy in real-world scenarios. These factors contribute to a certain degree of variability in radiological features, ultimately affecting the accuracy of the results. Although radiomics transforms traditional medical images into mineable data for a deeper and more objective analysis of the potential information within the images, inconsistencies may arise due to variations in CT scanners, imaging protocols, and the delineation of VOIs by different radiologists. Furthermore, the single-center and retrospective nature of the study may introduce selection bias and limit the generalizability of the results. To mitigate these issues, we selected radiomic features with good stability and employed various machine learning algorithms along with cross-validation. Our developed model could assist less experienced physicians when expert radiologists are absent or unavailable in resource-limited hospitals. Additionally, this model could serve as a primary reader for CT images of adrenal indeterminate nodules in lung cancer patients, thereby reducing the workload for radiologists. When significant differences are observed, it is essential to actively monitor or perform biopsies to confirm the diagnosis, thus aiding clinical practice in achieving precise staging of lung cancer and promoting individualized treatment.

In addition, although clinical staging of lung cancer was an independent risk factor of clinical-pathological features for distinguishing between adrenal metastatic nodules and benign nodules, the clinical-pathological-radiomics model did not include any clinical-pathological features. It was speculated that this may be related to a small sample size, a smaller contribution of clinical-pathological features to the comprehensive model, and a lower impact weight than radiomics features, which further confirmed the advantages of radiomics. Previous studies also found that the predictive performance of the comprehensive model may not be improved by combining radiomics features with clinical-pathological features or traditional imaging features (41, 53). However, this area warrants further investigation. Integrating these significant clinical-pathological features could potentially enhance the model’s diagnostic capability and should be explored in future research.

There were several limitations in this study: Firstly, our study was a single-center and retrospective analysis, which may lead to selection bias and limit the generalizability of the results. A future multi-center prospective study would provide a more diverse patient population and help to validate the findings across different clinical settings, thus enhancing the robustness and external validity of the developed models. Secondly, this study diagnosed some adrenal nodules according to imaging follow-up, which reflects and is also in line with the current clinical reality. Thirdly, the use of two different CT scanners and imaging protocols in this study may lead to variability in the radiomics features extracted, potentially impacting the performance of the model. Standardizing imaging protocols would reduce this variability and ensure consistent and reproducible radiomics feature extraction; However, it can be considered a strength of this study as it is more in line with the actual situation of Chinese healthcare and has a certain potential universality. Fourthly, our study only applied 3D VOI. Although previous studies reported that 3D VOI had a better ability to reflect tumor heterogeneity than 2D Region of interest (ROI) (54), 2D ROI had the advantage of being more convenient to operate, so it may be more feasible to use and easier to promote. Fifthly, the radiomics models constructed in this study had no external validation, and multi-center cooperation is needed to further improve the predictive performance and generalization ability of the models.

The combined radiomics model based on independent risk radiomics features of the plain and arterial CT images can non-invasively and efficiently predict adrenal metastatic nodules in lung cancer patients, and the predictive performance of which was significantly higher than the clinical-pathological-imaging model. In addition, the plain radiomics model, which also had high predictive ability, provided a convenient and accurate new method for predicting adrenal metastatic nodules in patients with contraindications for enhanced CT examination, effectively avoiding unnecessary further examinations.

## Data Availability

The raw data supporting the conclusions of this article will be made available by the authors, without undue reservation.
