# Supplementary material for: Adrenal indeterminate nodules: CT-based radiomics analysis of different machine learning models for predicting adrenal metastases in lung cancer patients
Source: Front Oncol. 2024 Nov 12;14:1411214. doi: 10.3389/fonc.2024.1411214 (PMC11588585; doi:10.3389/fonc.2024.1411214)
Supplement: Supplementary file 1 [file DataSheet1.docx]

Supplementary Material

Adrenal indeterminate nodules: CT-based radiomics analysis of different machine learning models for predicting adrenal metastases in lung cancer patients

Lixiu Cao^1+^, Haoxuan Yang^2+^, Huijing Wu^1+^, Hongbo Zhong^3^, Haifeng Cai^4^, Yixing Yu^5^, Lei Zhu^6^, Yongliang Liu^7^*and Jingwu Li^4^*

**Correspondence:**
Yongliang Liu;
e-mail: liuyongliang1974@126.com.

Jingwu Li;
e-mail: tslijingwu@163.com.

# Supplementary Figures and Tables

## Supplementary Figures

**
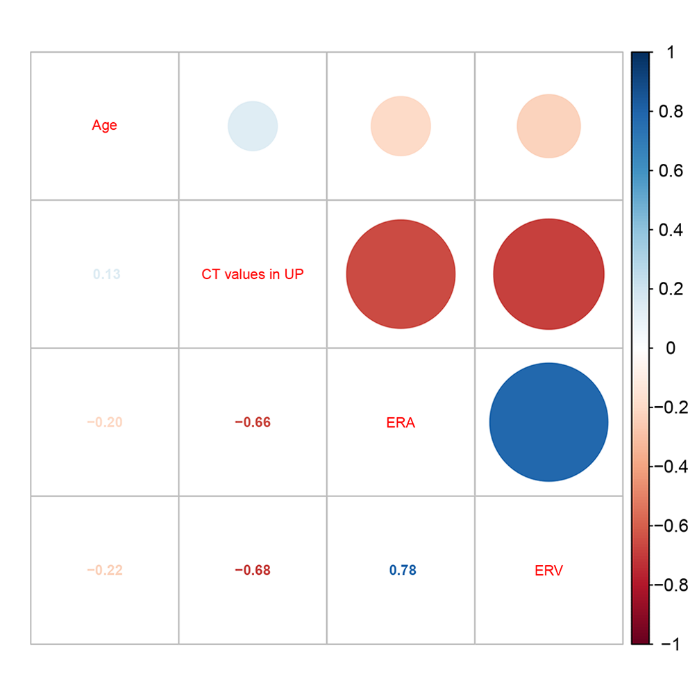
**

**Supplementary Figure 1** Strong correlation between arterial enhancement rate and portal enhancement rate.


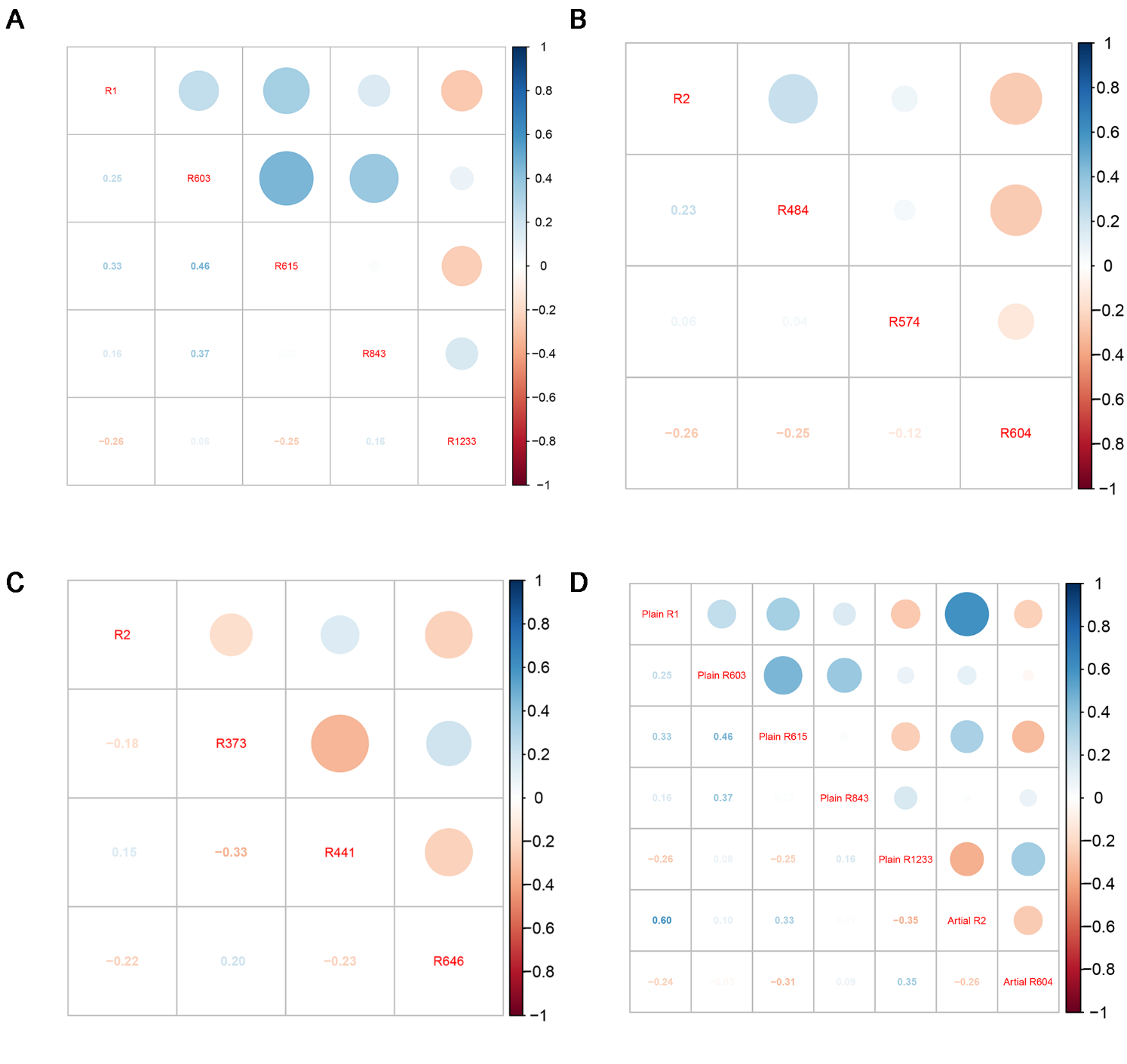


**Supplementary Figure 2** Radiomics heatmaps. (A)Heatmap depicting correlation coefficients matrix of five selected features in the plain phase. (B) Heatmap depicting correlation coefficients matrix of four selected features in the Arterial phase. (C) Heatmap depicting correlation coefficients matrix of four selected features in the Portal phase. (D) Heatmap depicting correlation coefficients matrix of seven selected features in the combined phase.

## Supplementary Tables

**Supplementary Table 1** Reproducibility between the two readers.

| Variables | ICC coefficient or Kappa value(95% CI) |
| --- | --- |
| Long diameter | 0.958(0.948-0.967) |
| Short diameter | 0.947(0.933-0.957) |
| The plain CT value | 0.986(0.982-0.989) |
| The arterial CT value | 0.928(0.907-0.945) |
| The portal CT value | 0.942(0.926-0.970) |
| Arterial enhancement rate | 0.951(0.937-0.962) |
| Portal enhancement rate | 0.949(0.934-0.960) |
| Location | 1.000 |
| Shape | 0.913 |
| Calcification | 1.000 |
| Cystic degeneration /necrosis | 0.977 |
| Peak enhancement phase | 0.812 |

**Supplementary Table 2** The ROC analysis of clinical-pathological and radiological individual features.

| Variables | Cutoff | AUC | Sensitivity | Specificity | Accuracy |
| --- | --- | --- | --- | --- | --- |
| Age | 52.5 | 0.626 | 0.872 | 0.375 | 0.639 |
| The plain CT values | 27.5 | 0.812 | 0.945 | 0.604 | 0.785 |
| Gender | - | 0.659 | 0.725 | 0.594 | 0.663 |
| Peak enhancement phase | - | 0.649 | 0.972 | 0.240 | 0.629 |
| Clinical stage of lung cancer | - | 0.713 | 0.927 | 0.406 | 0.683 |

**Supplementary Table 3** Comparison of AUCs between combined-phase radiomics models using different machine learning algorithms in the testing dataset

| Models | AUC | Z statistic | P |
| --- | --- | --- | --- |
| SVM model vs RF model | 0.938vs0.930 | 0.879 | 0.380 |
| SVM model vs LR model | 0.938vs0.924 | 1.448 | 0.148 |
| SVM model vs DT model | 0.938vs0.808 | 3.359 | <0.001 |
